# A kinetic metabolic study of lipid production in *Chlorella protothecoides* under heterotrophic condition

**DOI:** 10.1186/s12934-019-1163-4

**Published:** 2019-06-28

**Authors:** Xiaojie Ren, Jean-Sébastien Deschênes, Réjean Tremblay, Sabine Peres, Mario Jolicoeur

**Affiliations:** 1 Colin Ratledge Center for Microbial Lipids, School of Agriculture Engineering and Food Science, Shandong University of Technology, Zibo, China; 2 Research Laboratory in Applied Metabolic Engineering, Department of Chemical Engineering, École Polytechnique de Montreal, Centre-ville Station, P.O. Box 6079, Montreal, H3C 3A7 QC Canada; 3 Université du Québec à Rimouski, 310 allée des Ursulines, Rimouski, QC G5L 3A1 Canada; 4 LRI, Université Paris-Sud, CNRS, Université Paris-Saclay, 91405 Orsay, France; 5 MaIAGE, INRA, Université Paris-Saclay, 78350 Jouy-en-Josas, France

**Keywords:** Metabolic modelling, Kinetic model, Central carbon metabolism, Microalgae, *Chlorella protothecoides*, Dynamic flux analysis

## Abstract

**Background:**

Microalgae have been proposed as potential platform to produce lipid-derived products, such as biofuels. Knowledge on the intracellular carbon flow distribution may identify key metabolic processes during lipid synthesis thus refining culture/genetic strategies to maximize cell lipid productivity. A kinetic metabolic model simulating cell metabolic behavior and lipid production was first applied in the microalgae platform *Chlorella protothecoides* under heterotrophic condition. It combines both physiology and flux information in a kinetic approach. Cell nutrition, growth, lipid production and almost 30 metabolic intermediates covering central carbon metabolism were included and simulated.

**Results:**

Model simulations were shown to adequately agree with experimental data, which is suggesting that the proposed model copes with *Chlorella protothecoides* cells’ biology. The dynamic metabolic flux analysis using the model showed a reversible starch flux from accumulation to decomposing when glucose reached depletion, while net lipid flux shows a quasi-constant rate. The sensitive flux parameters on starch and lipid metabolism suggested that starch synthesis is the major competing pathway that affects lipid accumulation in *C. protothecoides*. Flux analysis also demonstrated that high lipid yield under heterotrophic condition is accompanied with high lipid flux and low TCA activity. Meanwhile, the dynamic flux distribution also suggests a relatively constant ratio of glucose distributed to biomass, lipid, starch, nucleotides as well as pentose phosphate pathway.

**Conclusion:**

The model described not only experimental data, but also unraveled intracellular carbon flow distribution and identify key metabolic processes during lipid synthesis. Most of the metabolic kinetics also showed statistical significance for metabolic mechanism. Therefore, this study unravels the mechanisms of the glucose impact on the dynamic carbon flux distribution, thus improving our understanding of the links between carbon fluxes and lipid metabolism in *C. protothecoides*.

**Electronic supplementary material:**

The online version of this article (10.1186/s12934-019-1163-4) contains supplementary material, which is available to authorized users.

## Background

Microalgae have been proposed as potential platform to produce lipid-derived products, such as biofuels. Previous studies have shown that most of microalgae cells accumulate lipids when the cell division is blocked or inhibited while carbon can still continue to be fixed (such as nitrogen shortage stress conditions), which results in reduced biomass growth and in tum constraints the total lipid yield [1–4]. Thus, there is a contradiction between lipid content and algae growth, which limits high lipid yield. Knowledge on the intracellular carbon flow distribution may identify key metabolic processes during lipid synthesis thus refining culture/genetic strategies to maximize cell lipid productivity [5–7]. The flux balance analysis (FBA) approach, which is based on pseudo steady-state approximation, has been applied to uncover the black-box of intracellular metabolism under steady state [8, 9]. It has been used in microalgae biosystems such as *Arghrospia platensis, Synechocystis* sp. PCC 6803 [10], *Chlamydomonas reinhardtii* [11, 12], *Chlorella protothecoides* [13] and *Chlorella* sp. [14]. For instance, in *Chlorella* sp., a shift in intracellular flux distribution was predicted during transition from nutrient sufficient phase to nutrient starvation phase of growth [14]. Another appealing modeling approach, which allows simulating a culture’s dynamics, is based on a kinetic transient-type approach [15, 16]. Such kinetic metabolic models describe cell dynamic behavior by inducing enzyme kinetics. An underestimated potential output of these kinetic metabolic models relies in their capacity to perform dynamic metabolic flux analysis from which key metabolic processes can be examined while assessing in silico hypothesis of genetic engineering and/or culture conditions management strategies [17]. However, to the best of our knowledge, kinetic metabolic model application in microalgae is relatively new. There are very few studies reporting dynamic metabolomics data of microalgae and even less on the development of mathematical models to describe cell metabolic dynamics [18].

In the present work, a kinetic metabolic model describing *Chlorella protothecoides* cellular metabolism was developed to describe heterotrophic culture mode. Unlike most dynamic models coping with algae physiology or steady-state metabolic level models (FBA), the current model combines both physiology and flux information in a kinetic approach. Cell growth, lipid production and almost 30 metabolic intermediates covering glycolysis, pentose phosphate pathway and TCA cycle and energetic metabolism were included and simulated. Multiple Michaelis–Menten equations are used to introduce metabolic kinetics of reaction rates and the Monod equation is used to describe the cell specific growth state, mass balances for each intermediate were considered in a dynamic profile. It can thus be used as an in silico platform for characterizing the cell lines as well as to search for ‘‘optimal’’ culture strategy through identify key metabolic processes during lipid synthesis.

## Materials and methods

### Algae stain and culture conditions

Details about algae species and culture conditions can be found in a previous work [19]. Briefly, *Chlorella protothecoides* (the Culture Collection of Alga at the University of Texas) culture in the dark was carried out in 2.8-L glass flasks with 10 g L^−1^ glucose as the carbon source and the modified basal medium (MBM), thus imposing a strict heterotrophic metabolism. Glucose concentration in the medium was analyzed by a biochemistry analyzer (YSI Life Science, 2700 select, Ohio, USA). Intracellular metabolites extraction was performed as described in previous work [19] and their quantification was carried out by UPLC/MS/MS system (1290 model, Agilent Technologies, Santa Clara, CA, USA), starch analysis was performed using a starch assay kit (Sigma-Aldrich, St. Louis, MO, USA). Total lipid quantification was done according to Drochiou’s method and described in previous work [19].

### Model development

#### Model structure

**Fig. 1:**
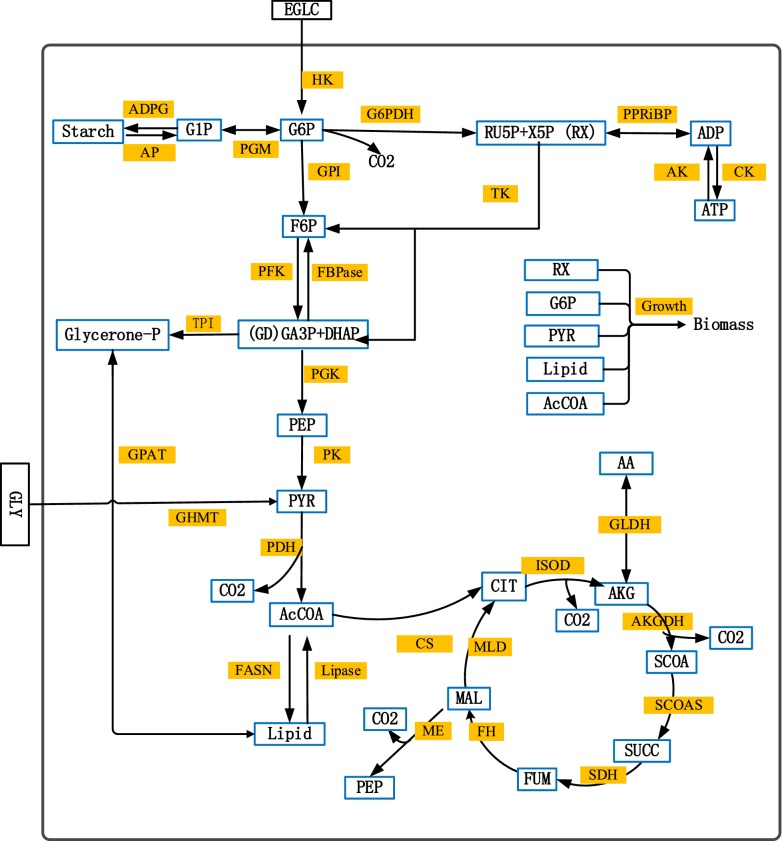
The model metabolic network for heterotrophic *Chlorella protothecoides*

A kinetic metabolic model was developed to describe the central carbon metabolism of a microalgae platform, including glycolysis, TCA (tricarboxylic acid) cycle, pentose phosphate pathway, total lipid synthesis, starch synthesis, amino acids metabolism, energy metabolism and biomass synthesis. The metabolic network (Fig. 1) was first built according to databases such as KEGG, MetaCyc, DiatomCyc, BioCyc as well as from literature [18, 20, 21]. In this work, *Chlorella protothecoides* cells were considered as a unique compartment with no specific intracellular compartments such as mitochondria, chloroplast, vacuoles, vesicles and nucleus. Energy metabolism was considered as a global reaction where de novo synthesis and substrate level phosphorylation were combined in a unique pathway. Reversible reactions involving storage carbon such as starch and lipid catabolism were described. The stoichiometry of the biochemical reactions of the network is based on the flux balance analysis on *Chlorella protothecoides* [18]. A full list of the model reactions and reactions stoichiometry is listed in Table 1.

**Table 1:** Reactions of a metabolic network

No.	Enzyme	Description	Reaction
1	HK	Hexokinase	EGLC ⇒ G6P
2	GPI	Glucose 6 phosphate isomerase	G6P ⇒ F6P
3	PFK	6 phosphofructokinase	F6P ⇒ 2 GD
4	FBPase	Fructose biphosphate aldolase	2 GD ⇒ F6P
5	PGK	Phosphoglycerate kinase	GD ⇒ PEP
6	PK	Pyruvate kinase	PEP ⇒ PYR
7	PDH	Pyruvate dehydrogenase	PYR ⇒ AcCOA + CO_2_
8	FASN	Fatty acid synthase	12 AcCOA ⇒ Lipid
9	Lipase	Lipase	Lipid ⇒ 12 AcCOA
10	GPAT	Glycerol-3-phosphate acyltransferases	GlyP = Lipid
11	TPI	Triosephosphate isomerase	GD ⇒ GlyP
12	G6PDH	Glucose 6 phosphate 1 dehydrogenase	G6P ⇒ RX + CO_2_
13	TK	Transketolase	3 RX ⇒ 2 F6P + GD
14	PPRiBP	Phosphoribosyl-diphosphate synthetase	RX = ADP
15	CK	Creatine kinase	ADP ⇒ ATP
16	AK	Adenylate kinase	ATP ⇒ ADP
17	PGM	Phosphoglucomutase	G6P = G1P
18	ADPG	Adenosine diphosphate glucose-starch glucosyltransferase	25 G1P ⇒ Starch
19	AP	Amylase	Starch ⇒ 25 G1P
20	GHMT	Glycine hydroxymethyltransferase	GLY ⇒ PYR
21	CS	Citrate synthase	AcCOA ⇒ CIT
22	MLD	Malate dehydrogenase	MAL ⇒ CIT
23	ISOD	Isocitrate dehydrogenase	CIT ⇒ AKG + CO_2_
24	AKGDH	Oxoglutarate dehydrogenase	AKG ⇒ SCOA + CO_2_
25	GLDH	Glutamate dehydrogenase	AKG = AA
26	SCOAS	Succinyl CoA ligase	SCOA ⇒ SUCC
27	SDH	Succinate dehydrogenase	SUCC ⇒ FUM
28	FH	Fumarate hydratase	FUM ⇒ MAL
29	ME	Malic enzyme	MAL ⇒ PEP + CO_2_
30	Growth	Biomass synthesis	G6P + RX + PYR + Lipid + AcCOA ⇒ X

‘ ⇒’, represents unidirectional reactions; =’, represents reversible reactions

The Michaelis–Menten kinetic equation is used to describe each flux rate (Table 2). The cells specific growth rate (equation no. 30 in Table 2), accounting for biomass synthesis from precursors of the major cell constituents such as RX (R5P and X5P), G6P, PYR, AcCOA and total lipid. RX is normally used to synthesize nucleotides, DNA and RNA; G6P leads to organic phosphates providing energy for maintenance and metabolism; PYR is feeding amino acids metabolism which leads to protein formation; AcCOA is the precursor of fatty acids; while the lipid pool is the main contributor to cell mass accumulation in the algae platform.

**Table 2:** Kinetic equations of the metabolites fluxes in the model

No.	Kinetic equations
1	$V_{HK} = V_{{{ \text{max} }\_HK}} *\frac{EGLC}{{K_{m\_HK\_EGLC} + EGLC}}$
2	$V_{GPI} = V_{{{ \text{max} }\_GPI}} *\frac{G6P}{{K_{m\_GPI\_G6P} + G6P}}$
3	$V_{PFK} = V_{{{ \text{max} }\_PFK}} *\frac{F6P}{{K_{m\_PFK\_F6P} + F6P}}$
4	$V_{FBPase} = V_{{{ \text{max} }\_FBPase}} *\frac{GD}{{K_{m\_FBPase\_GD} + GD}}$
5	$V_{PGK} = V_{{{ \text{max} }\_PGK}} *\frac{GD}{{K_{m\_PGK\_GD} + GD}}$
6	$V_{PK} = V_{{{ \text{max} }\_PK}} *\frac{PEP}{{K_{m\_PK\_PEP} + PEP}}$
7	$V_{PDH} = V_{{{ \text{max} }\_PDH}} *\frac{PYR}{{K_{m\_PDH\_PYR} + PYR}}$
8	$V_{FASN} = V_{{{ \text{max} }\_FASN}} *\frac{AcCOA}{{K_{m\_FASN\_AcCOA} + AcCOA}}$
9	$V_{Lipase} = V_{{{ \text{max} }\_Lipase}} *\frac{Lipid}{{K_{m\_Lipase\_Lipid} + Lipid}}$
10	$V_{GPAT} = V_{{{ \text{max} }\_GPAT}} *\frac{GlyP}{{K_{m\_GPAT\_GlyP} + GlyP}} - V_{{{\text{maxr}}\_GPAT}} *\frac{Lipid}{{K_{m\_GPAT\_Lipid} + Lipid}}$
11	$V_{TPI} = V_{{{ \text{max} }\_TPI}} *\frac{GD}{{K_{m\_TPI\_GD} + GD}}$
12	$V_{G6PDH} = V_{{{ \text{max} }\_G6PDH}} *\frac{G6P}{{K_{m\_G6PDH\_G6P} + G6P}}$
13	$V_{TK} = V_{{{ \text{max} }\_TK}} *\frac{RX}{{K_{m\_TK\_RX} + RX}}$
14	$V_{PPRiBP} = V_{{{ \text{max} }\_PPRiBP}} *\frac{RX}{{K_{m\_PPRiBP\_RX} + RX}}$
15	$V_{CK} = V_{{{ \text{max} }\_CK}} *\frac{ADP}{{K_{m\_CK\_ADP} + ADP}}$
16	$V_{AK} = V_{{{ \text{max} }\_AK}} *\frac{ATP}{{K_{m\_AK\_ATP} + ATP}}$
17	$V_{PGM} = V_{max\_PGM} *\frac{G6P}{{K_{m\_PGM\_G6P} + G6P}} - V_{maxr\_PGM} *\frac{G1P}{{K_{m\_PGM\_G1P} + G1P}}$
18	$V_{ADPG} = V_{{{ \text{max} }\_ADPG}} *\frac{G1P}{{K_{m\_ADPG\_G1P} + G1P}}$
19	$V_{AP} = V_{{{ \text{max} }\_AP}} *\frac{Starch}{{K_{m\_AP\_Starch} + Starch}}$
20	$V_{GHMT} = V_{{{ \text{max} }\_GHMT}} *\frac{GLY}{{K_{m\_GHMT\_GLY} + GLY}}$
21	$V_{CS} = V_{{max_{\_} CS}} *\frac{AcCoA}{{K_{m\_CS\_AcCoA} + AcCoA}}$
22	$V_{MLD} = V_{max\_MLD} *\frac{MAL}{{K_{m\_MLD\_MAL} + MAL}}$
23	$V_{ISOD} = V_{max\_ISOD} *\frac{CIT}{{K_{m\_ISOD\_CIT} + CIT}}$
24	$V_{AKGDH} = V_{{{ \text{max} }\_AKGDH}} *\frac{AKG}{{K_{m\_AKGDH\_AKG} + AKG}}$
25	$V_{GLDH} = V_{{{ \text{max} }\_GLDH}} *\frac{AKG}{{K_{m\_GLDH\_AKG} + AKG}} - V_{{{\text{maxr}}\_GLDH}} *\frac{AA}{{K_{m\_GLDH\_AA} + AA}}$
26	$V_{SCOAS} = V_{{{ \text{max} }\_SCOAS}} *\frac{SCOA}{{K_{m\_SCOAS\_SCOA} + SCOA}}$
27	$V_{SDH} = V_{{{ \text{max} }\_SDH}} *\frac{SUCC}{{K_{m\_SDH\_SUCC} + SUCC}}$
28	$V_{FH} = V_{{{ \text{max} }\_FH}} *\frac{FUM}{{K_{m\_FH\_FUM} + FUM}}$
29	$V_{ME} = V_{{{ \text{max} }\_{\text{ME}}}} *\frac{MAL}{{K_{m\_ME\_MAL} + MAL}}$
30	$V_{growth} = V_{{{ \text{max} }\_growth}} *\frac{G6P}{{K_{{m\_growth_{G6P} }} + G6P}} *\frac{RX}{{K_{{m\_growth_{RX} }} + RX}} *\frac{PYR}{{K_{{m\_growth_{PYR} }} + PYR}}*\frac{AcCOA}{{K_{m\_growth\_AcCOA} + AcCOA}} *\frac{Lipid}{{K_{m\_growth\_Lipid} + Lipid}}$

For each metabolite in the model, a mass balance equation (Eq. 1) included the sum of all the input and output fluxes minus cell dilution effect from the cell division phenomenon. The specific consumption of precursors to cell mass synthesis was also considered in the precursors’ mass balances.

1 $$\frac{{\left. {d[M_{i} } \right]}}{{{\text{d}}t}} = \left( {\mathop \sum \limits_{m = 1}^{a} V_{input_i} - \mathop \sum \limits_{n = 1}^{b} V_{output_j} - \left[ {M_{i} } \right] \times V_{growth} - c_{i} \times V_{growth} } \right)$$

where *M*_*i*_ is the concentration of each metabolite at time t, *a* is the input flux number, *b* is the output flux number, *V*_*input*_ and *V*_*output*_ are the flux rates at each metabolite node. $c_{i}$ is the stoichiometric coefficient for biomass precursors, *V*_*growth*_ is the specific growth rate. So $\left[ {M_{i} } \right] \times V_{growth}$ is the cell dilution term and $c_{i} \times V_{growth}$ is the growth contribution term for biomass precursors (G6P, RX, PYR, Lipid, AcCOA).

All the ordinary differential equations of metabolites were provided as in Additional file 1: Table S1. The ordinary equation for biomass was described by $\frac{dX}{dt} = V_{growth} *X$, listed in Additional file 1: Table S1 (Eq. 22). Where $V_{growth}$ is the specific growth rate, *X* is the biomass concentration. The cell specific growth rate $V_{growth}$ was described by Eq. 30 in Table 2. A program generating automatically the Matlab code and differential equations for a specific metabolic network of reactions was developed and used (S. Peres and M. Jolicoeur, unpublished). The ordinary differential equations system were performed using Matlab (the MathWorks Inc., Natick, MA, USA) with the “ode23” solver to get the model simulations result.

#### Model parameters estimation

The model has 77 parameters, which include 34 maximum flux rates, 38 enzyme half-saturated constants, and 5 growth coefficients for the 5 growth precursors contributing to biomass synthesis. Initial metabolite concentrations (i.e. at t = 0) were taken from experimental data, which include 24 intracellular metabolites distributed in 8 pathways covering 30 metabolic reactions [19] or from literature (Table 3).

**Table 3:** State variables description and initial conditions

No.	Metabolites	Description	Values	Units
1	ADP	Adenosine diphosphate	2.22E−03	mmol/gDW
2	AKG	a-Ketoglutarate	5.88E−05	mmol/gDW
3	ATP	Adenosine triphosphate	1.43E−02	mmol/gDW
4	AcCOA	Acetyl–coenzyme A	2.56E−04	mmol/gDW
5	CIT	Citrate	2.00E−04	mmol/gDW
6	F6P	Fructose 6-phosphate	4.72E−05	mmol/gDW
7	FUM	Fumarate	2.42E−05	mmol/gDW
8	G1P	Glucose 1-phosphate	1.05E−05	mmol/gDW
9	G6P	Glucose 6-phosphate	2.36E−04	mmol/gDW
10	GD	Glyceraldehyde 3-phosphate and dihydroxyacetone phosphate	1.94E−04	mmol/gDW
11	AA	Amino acids	3.80E+01	mmol/gDW
12	GlyP	Glycerone-phosphate	2.00E−04	mmol/gDW
13	Lipid	Lipid	4.76E−01	mmol/gDW
14	MAL	Malate	1.39E−04	mmol/gDW
15	PEP	Phosphoenolpyruvate	2.37E−05	mmol/gDW
16	PYR	Pyruvate	1.10E−04	mmol/gDW
17	RX	Ribose 5-phosphate and xylose-5-phosphate	2.26E−05	mmol/gDW
18	SCOA	Succinyl–coA	2.00E−05	mmol/gDW
19	SUCC	Succinate	2.00E−05	mmol/gDW
20	Starch	Starch	4.45E−03	mmol/gDW
21	EGLC	Extracellular glucose	5.53E+01	mmol/L
22	X	Biomass	4.21E−02	gDW/L
23	GLY	Glycine	1.32E+00	mmol/L
24	CO_2_	Carbon dioxide	1.20E−04	mmol/L

Initial kinetic parameter values (*V*_*max*_, *K*_*m*_) for each flux and enzymes in the model were taken within ranges found in the enzyme database BRENDA (http://www.brenda-enzymes.org), and the respective units (mmol/L) were converted to comply with the model (mmol/gDW) by dividing 10 gDW/L biomass obtained in our culture. First estimates of maximal flux rates (*V*_*max*_) have been calculated from experimental data [19], or from BRENDA. Model parameter values were determined following the method proposed in Rizzi et al. [22]. Briefly, the time course of each metabolite with experimental concentration data were defined as fixed mathematical functions, enabling the procedure for parameter values optimization to focus first on non-measured metabolites. In the present case of a high number of parameters, this approach allows accelerating parameter values identification. An objective function (Eq. 2), defined as the weighted sum of squared residues between experimental data and simulated values for each metabolites *m* at time *k*, where the weight is the experimental data for each state variable, was used to quantify simulation error.

2 $${ \text{min} }\left( {\mathop \sum \limits_{i = 1}^{N} \mathop \sum \limits_{t = 1}^{T} \left( {\frac{{M_{i,t}^{exp} - M_{i,t}^{sim} }}{{M_{i,t}^{exp} }}} \right)^{2} } \right)$$

Based on this objective function, a sensitivity analysis of model parameters was performed to identify the sensitive ones in order to avoid over-parameterization, by then keeping constant non-sensitive parameters. Sensitivity analysis was performed by changing each parameter from − 70 to + 150% one at a time while holding others constant. From the Matlab optimization toolbox, the “linsqurfit” sub-routine was used to identify model parameter values. This process of parameter calibration was continued until minimizing the objective function, i.e. the simulated results closely following experimental data. Final parameter values of the model are shown in Table 4. Confidence intervals of estimated parameters were evaluated using the Matlab sub-routine “nlparci.m” (Table 4). It is clear there is no unique solution for parameter values in such an underdetermined system.

**Table 4:** Parameter values and 95% confidence intervals of the highly sensitive parameters

Parameters	Values	Confidence interval	Parameters	Values	Confidence interval
V_max_HK_	20	(19.989, 20.011)	km_HK_EGLC	0.8	
V_max_GPI_	17	(16.998, 17.002)	km_GPI_G6P	0.0001	(8.37E−5, 1.16E−4)
V_max_PFK_	23	(22.998, 23.002)	km_PFK_F6P	0.00003	
V_max_FBPase_	1	(0.008, 1.003)	km_PGK_GD	0.000004	(3.12E−6, 4.88E−6)
V_max_PK_	37	(36.991, 37.009)	km_PK_PEP	0.000007	
V_max_PDH_	40	(39.997, 40.003)	km_PDH_PYR	0.00001	
V_max_G6PDH_	9		km_G6PDH_G6P	0.0009	
V_max_PGK_	30	(29.997, 30.003)	km_TK_RX	0.0001	
V_max_TK_	13	(12.080, 13.041)	km_ADPG_G1P	0.00005	
V_max_ADPG_	10	(9.987, 10.008)	km_AP_Starch	0.008	
V_max_AP_	2	(1.080, 2.023)	km_PGM_G6P	0.00004	
V_max_PGM_	16	(15.992, 16.008)	km_PGM_G1P	0.000005	
V_maxr_PGM_	14	(13.995, 14.005)	km_GHMT_GLY	0.1	
V_max_GHMT_	6	(5.032, 6.103)	km_growth_G6P	0.0000001	(3.20-E−6, 3.40E−6)
V_max_growth_	2	(1.076, 2.045)	km_growth_PYR	0.0000001	(2.47E−7, 4.47E−7)
V_max_PPRiBP_	15		km_growth_RX	0.0000006	(4.46E−7, 7.54E−7)
V_maxr_PPRiBP_	7		km_growth_Lipid	0.001	(8.16E−4, 1.18E−3)
V_max_CK_	5		km_growth_AcCOA	0.000001	
V_max_AK_	4.5		km_FBPase_GD	0.0003	(0.0002, 0.0004)
V_max_TPI_	0.01		km_PPRiBP_RX	0.00001	
V_max_GPAT_	0.01		km_PPRiBP_ADP	0.005	
V_maxr_GPAT_	0.06		km_CK_ADP	0.001	
V_max_FASN_	31.3		km_AK_ATP	0.002	
V_max_Lipase_	12.12		km_GPAT_GlyP	0.0001	
V_max_CS_	2		km_GPAT_Lipid	0.01	(0.0098, 0.0102)
V_max_ISOD_	0.3		km_FASN_AcCOA	0.002	
V_max_AKGDH_	0.3		km_Lipase_Lipid	0.01	(0.0099, 0.0101)
V_max_SCOAS_	1		km_TPI_GD	0.002	
V_max_SDH_	13		km_CS_AcCOA	0.2	
V_max_FH_	3		km_GLDH_AA	3	
V_max_MLD_	0.1		km_GLDH_AKG	3	
V_max_ME_	0.1		km_ISOD_CIT	0.7	
V_max_GLDH_	0.01		km_AKGDH_AKG	0.007	
V_maxr_GLDH_	0.02		km_SCOAS_SCOA	0.001	
V_growth_RX	0.001		km_SDH_SUCC	0.00002	
V_growth_PYR	0.002		km_FH_FUM	0.008	
V_growth_G6P	0.001		km_MLD_MAL	0.003	
V_growth_Lipid	0.001	(0.0008, 0.001)	km_ME_MAL	0.001	
V_growth_AcCOA	0.008				

The flux-rates’ units are in mmol gDW^−1^ day^−1^, except for the maximum specific growth rate *(V*_*max, growth*_*)* which is in day^−1^. Enzymes affinity constants’ units are in mmol gDW^−1^, except for HK and GHMT, which are in mmol L^−1^

## Results and discussion

### Model simulates algae cell behavior under heterotrophic condition

**Fig. 2:**
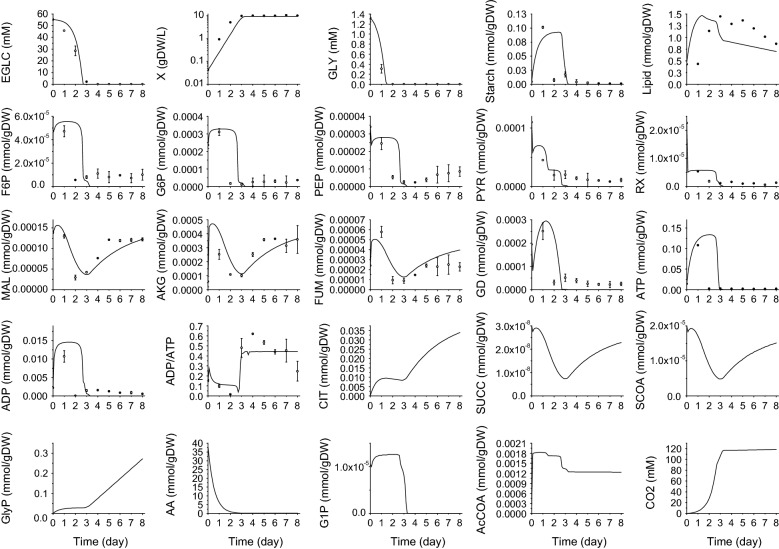
Simulation result versus experimental data for *Chlorella protothecoides* heterotrophic behavior (experimental data: open squares, simulated data: solid line. Experimental data were taken from a previous work [19], where the error bars represent the standard error of triplicates data.)

The final calibrated model adequately simulates the experimental data (Fig. 2). Cell growth, as well as extracellular metabolites such as glucose and glycine are closely simulated. More importantly, total lipids and starch as the main products were also simulated adequately. The model simulation also followed closely the dynamics of intracellular metabolites, which distributed in glycolysis, PPP pathway, TCA cycle as well as energy metabolism. These results thus confirm the model structure as well as its calibrated kinetic parameters to simulate algae cells metabolism and products accumulation dynamics. Indeed, in this work, both the experimental data and model simulations show glycolysis and PPP pathways being more affected by glucose supply while TCA metabolism, which is fed by both carbon and nitrogen metabolisms, seems more robust to perturbations such as extracellular glucose depletion. The intracellular nutrition storage pool in the form of TCA cycle metabolites seemed to maintain biomass in the later growth phase. From both simulation and experimental data, algae biomass still accumulates while glycine and other amino acids pool (AA) reached values under the detection limits. However, this phenomenon was only observed for nitrogen sources since cell biomass growth stopped simultaneously to glucose depletion. This intracellular nutrients management phenomenon has also been modeled and proved in phytoplankton and plant cells [23]. Where the model premises were based on observations that cell growth continued after the exhaustion of external nitrogen pool, being then supported by the consumption of intracellular nitrogen pools such as chlorophyll molecules.

### Dynamic metabolic flux analysis reveals lipid and cellular metabolic behavior in *Chlorella protothecoides*

**Fig. 3:**
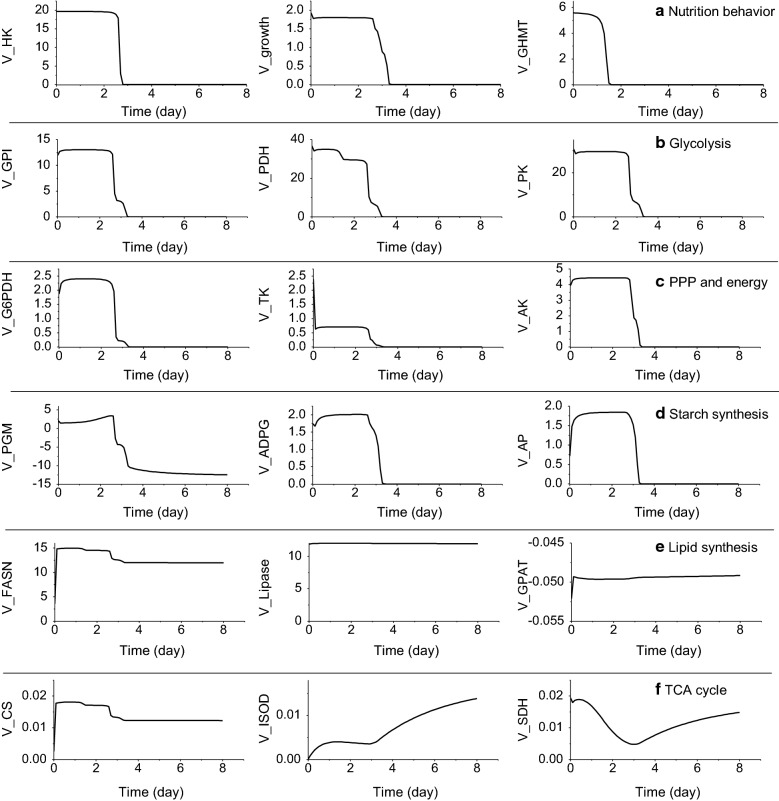
Flux rates of all the reactions in the model system. **a** Nutrition fluxes and growth rate; **b** glycolysis fluxes; **c** PPP pathway fluxes; **d** starch synthesis fluxes; **e** lipid synthesis fluxes; **f** TCA cycle fluxes. All flux units were in mmol gDW^−1^ day^−1^

Considering all the above, it is thus clear that the model structure allows simulating heterotrophic *Chlorella protothecoides* cell behavior. The model was then taken as an in silico tool and perform a dynamic metabolic flux analysis estimating flux distribution. A dynamic metabolic flux analysis was performed from model simulation (Fig. 3). Looking at glucose flux (*V*_*HK*_), glycine flux (*V*_*GHMT*_) as well as cell specific growth rate (*V*_*growth*_) (Fig. 3a), it is clear that cell growth proceeds simultaneously to carbon source uptake, but not proportionally to nitrogenous source uptake. Interestingly and as previously discussed for glycine concentration, glycine flux (*V*_*GHMT*_) ceased more than 24 h prior to growth cessation. Fluxes of PPP pathway and starch synthesis (Fig. 3c, d) originate from G6P are partially affected in some extent by glucose flux (Fig. 3a). For instance, model simulation *V*_*PGM*_ flux showed being reversible from accumulation to decomposing at around day 3, where glucose reached depletion. This suggests that starch, which is an intracellular carbon storage pool, rapidly responds to a low carbon source level threshold, contributing providing continuous carbon flow feeding cell metabolism and maintenance. However, as an alternative carbon storage pool, net lipid flux shows a quasi-constant rate, composed of a synthesis flux (*V*_*FASN*_) that was slightly affected at glucose depletion and two catabolic fluxes (*V*_*Lipase*_ and *V*_*GPAT*_) which stayed quite constant (11.87–12.04 mmol gDW^−1^ day^−1^ and 0.05 mmol gDW^−1^ day^−1^ respectively) (Fig. 3e). Interestingly, TCA cycle fluxes (*V*_*ISOD*_*, V*_*SDH*_) (Fig. 3f) exhibited a minimum value at glucose depletion, for increasing thereafter. As previously mentioned, the TCA cycle is closely related to carbon and nitrogen metabolism, it seems after carbon source depletion, some carbon and nitrogen dependent compounds (such as pigment) stopped synthesis, which squeezed the intracellular nitrogen source flux goes to TCA cycle. As in heterotrophic culture, *C. protothecoides* represents yellowish because of carotene content is higher than chlorophyll [24]. However, after glucose depletion, we found the color of culture turns from yellow to green, and the carotene gets to degrade. Moreover, CS flux dynamics closely follows the lipid synthesis flux although it’s quite low compared with lipid synthesis. As CS is competing the same substrate from FASN, the flux of these two enzymes are quite dependent on the concentration of AcCOA, which is in agreement from model prediction.

**Fig. 4:**
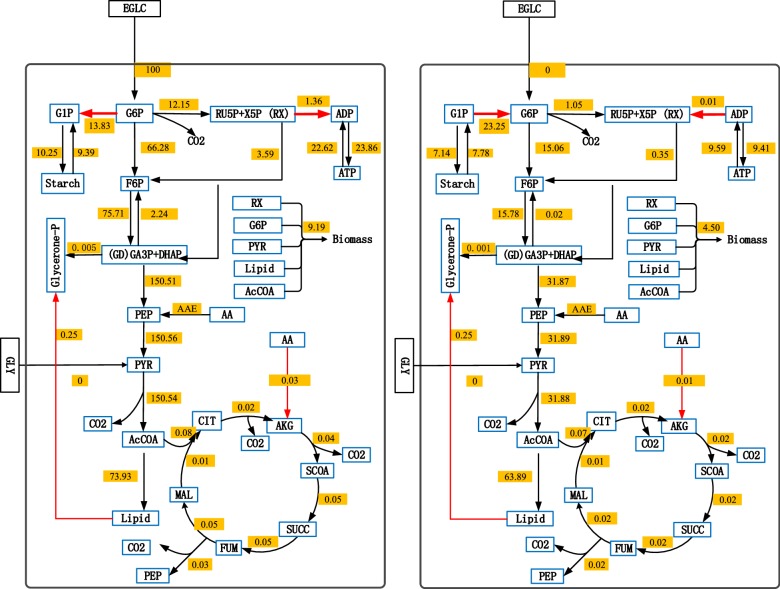
Flux distribution under heterotrophic cultivation at exponential phase (48 h) and lipid peak time point (72 h). All the flux values were normalized to an uptake flux of 100 mmol g^−1^DW h^−1^ glucose. Red arrows represents the flux direction for the four reversible reactions

A closer view of flux rates were estimated at 48 h before glucose depletion in the exponential growth phase. For comparison purposes, all the flux values were normalized to an uptake flux of 100 mmol g^−1^DW h^−1^ glucose (Fig. 4). Flux results agree with that reported in the previous reports [13, 18]. For example, in [18], who performed a flux balance analysis at steady state for *Chlorella* sp. under heterotrophic condition, with a GPI flux of 66.28 mmol g^−1^ DW h^−1^ (leading to glycolysis), G6PDH of 12.15 (leading to PPP pathway) and PGM of 13.83 (leading to starch), compared to 49.8 mmol g^−1^ DW h^−1^, 32.04 and 17.3 respectively in our work. The total flux to G6P obtained from our model is of 92.26 mmol g^−1^ DW h^−1^ compared that of 99.22 in literature. The net flow From F6P to GD (PFK minus FBPase) was of 73.47 mmol g^−1^DW h^−1^ compared to 70.35 mmol g^−1^ DW h ^−1^ (from F6P to GAP), and the flux from GD to PEP was of 150.51 mmol g^−1^ DW h^−1^ in our model versus 148.25 (from GAP to PEP) in literature. The fluxes of nucleotides synthesis (from RX to ADP) was of 1.36 mmol g^−1^DW h^−1^ compared to 0.67 mmol g^−1^DW h^−1^ (from PRPP to DNA and RNA). Biomass synthesis rate was of 9.19 compared to 7.36 in [18].

Furthermore, downstream fluxes to AcCOA, the sum of the downstream lipid and TCA cycle flux was of 74.01 mmol g^−1^DW h^−1^ compared to 86.14 g^−1^DW h^−1^ in literature. Although a similar total flux around the TCA cycle was obtained, with 73.93 mmol g^−1^DW h^−1^ at lipid branch and 0.08 at TCA branch, different results were reported in [18] with 81.21 mmol g^−1^DW h^−1^ at TCA cycle and 4.82 mmol g^−1^DW h^−1^ at lipid branch. This discrepancy may rely on a high lipid level (13.13% DW) in our cell culture compared to that in [18] (1% DW). Differences in culture conditions may be involved as well. Meanwhile, this difference of high lipid synthesis flux and low TCA cycle fluxes were also found in Wu’s work, where a ^13^C metabolic flux analysis was accompanied with flux balance analysis in *Chlorella protothecoides* [13]. Thus, from both the dynamic and steady state flux analysis, high lipid content in *Chlorella* is mainly due to low TCA split-flow. In our result, lipid was fully accumulated at 72 h, so the metabolic fluxes at 72 h was also extracted from the dynamic flux profile. Along with the glucose deleption, most of the metabolic fluxes decreased in different extent, some even decreased up to 78.87% (EMP pathway fluxes). However, the re-arrangement of flux distribution from starch and energy catabolism to lipid synthesis was obvious. At 48 h, starch and ATP were accumulating (*V*_*PGM*_ is 13.83 mmol g^−1^DW h^−1^) along with glucose assimilation. However, after glucose deleption at 72 h, starch was catablising (*V*_*PGM*_ is − 23.25 mmol g^−1^DW h^−1^) as a storage pool and ATP was also consuming (*V*_*PPRiBP*_ is − 0.01 mmol g^−1^DW h^−1^) to maintain other metabolism. Lipid synthesis flux (*V*_*FASN*_), decreased a little bit from 73.93 to 63.89 mmol g^−1^DW h^−1^ (decreased 13.51%), which was less impacted by glucose deleption compared with other fluxes on EMP pathway. This may due to a constant feeding flux converting from starch and ATP catabolism. Therefore, the energy stored in starch and ATP seams to be converted to a more stable storage pool as lipid after glucose deleption.

**Fig. 5:**
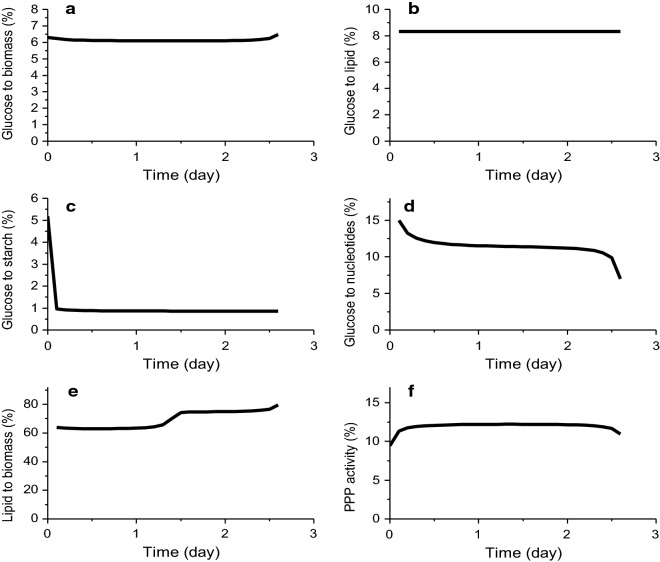
Metabolic flux ratios between different pathways. **a** Glucose contribution to biomass, **b** to lipids, **c** to starch and **d** to nucleotides; **e** lipid contribution to biomass; **f** PPP pathway activity

We also looked at the major carbon distribution before glucose depletion. Model simulations show that the glucose uptake rate (*V*_*HK*_) and the glycolytic fluxes went down to a very low level after day 2.6, we have thus analyzed their related flux ratios only before glucose depletion (< 2.6 days). First, we evaluated that 6% of the glucose flux contributes to biomass synthesis and growth (*V*_*growth*_-to-*V*_*PK*_ ratio) (Fig. 5a), a value comparable to the literature with 3.9% [25]. Within the same range, 8% (8.323–8.325%) of the glucose flux feed lipid synthesis (*V*_*FASN*_–*V*_*Lipase*_ to *V*_*PDH*_ ratio) (Fig. 5b). However, as a main product contributing to biomass, the lipid catabolism-to-biomass synthesis and growth ratio (*V*_*FASN*_ –*V*_*Lipase*_ –*V*_*GPAT*_ to *V*_*growth*_) shows two successive constant values at around 60% increasing at 80% at mid-exponential growth phase (1.5 d) (Fig. 5e). Model simulations also suggest that around 1% of the glucose flux goes to starch synthesis (*V*_*PGM*_ to *V*_*HK*_) (Fig. 5c), and that 15% to 7% of the glucose flux feed nucleotides synthesis (*V*_*PPRiBP*_ to *V*_*G6PDH*_) (Fig. 5d). Concerning the PPP pathway activity, around 12% of the glucose uptake flux flow into the pentose phosphate pathway (*V*_*G6PDH*_ to *V*_*HK*_) (Fig. 5f). Therefore, the dynamic metabolic flux analysis give our lights of the carbon distribution in *Chlorella protothecoides*.

### Parameter sensitivity showed biological significance on metabolic kinetics

**Fig. 6:**
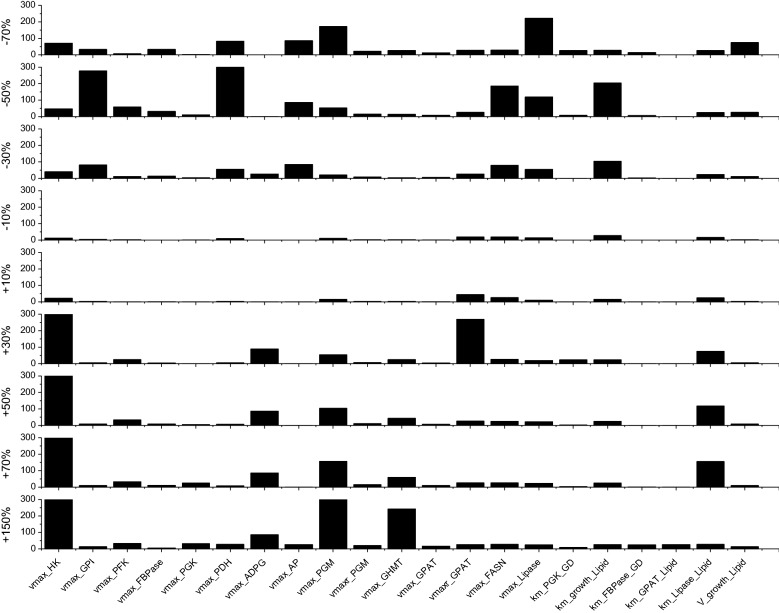
Sensitivity analysis on model parameters. Vertical axis value represents percentage change in the objective function for parameter change from − 70 to + 150% around the optimized value. Parameters not shown have percentage changes less than 10%

A sensitivity analysis on model parameters showed flux maximum rate constants (*V*_*max,i*_) to be more sensitive than affinity constants (*K*_*m,i*_). For the final calibrated model 21 parameters, 15 maximum flux rates and 6 enzyme affinity constant (Fig. 6), out of 77 revealed greater sensitivity, defined as affecting the objective function of more than 10% when applying a − 70% to + 150% parameter value change around its optimized value.

The most sensitive parameters are *V*_*max,HK*_ and *V*_*max,GHMT*_, which are both at the entrance of the major carbon and nitrogen sources; *V*_*max,GPI*_, *V*_*max,PGM*_ and *V*_*max,PDH*_, which refer to fluxes at the intersection of glycolysis, starch and lipid metabolisms are also highly sensitive, while PPP pathway (*V*_*max,TK*_) and TCA cycle parameters, showed a low sensitivity level. Since the major intersections from glycolysis including starch synthesis, PPP pathway which finally leads to nucleic acids, and TCA cycle which is related to protein synthesis, and the fatty acids synthesis which leads to lipids. The sensitive flux parameters on starch and lipid metabolism suggested that starch synthesis is the major competing pathway that affect lipid accumulation in *C. protothecoides*. It has also been reported in *Chlamydomonas reinhardtii*, when starch biosynthesis is blocked (sta6 mutant), the lipid content could be greatly boost, some can reach up to 30-fold [26]. Except for starch flux sensitivity, *V*_*max,FASN*_, *V*_*max,Lipase*_ and *V*_*max,GPAT*_ are also sensitive. As *V*_*max,FASN*_ and *V*_*max,Lipase*_ are related to lipid synthesis and degradation, they are responsible for the balance of cellular lipid pool. Meanwhile, *V*_*max,GPAT*_ is in charge of providing glycerone-phosphate as the neutral lipid skeleton. The sensitivity of these fluxes gave us light on the genetic strategy for lipid yield promotion. Interestingly, there are two highly sensitive affinity constants (*km*_*,growth_lipid*_ and *km*_*,Lipase_lipid*_), referring to the importance of lipid for cell biomass growth. Algae cell is a great platform accumulating lipids, some algae species could accumulate lipids up to 70% of their biomass. In *C. protothecoides*, the lipid content could reach 36% under heterotrophic condition, although in our work lipid content only reached 13% DCW, the sensitive of lipid affinity constant to growth suggest a huge potential to optimize the enzyme activity. Some reactions or pathways (i.e. their kinetic parameters) such as the maximum specific growth rate, PPP pathway and TCA cycle, showed a low sensitivity level, which suggest these are robust pathways. Final parameter values and the 95% confidence intervals for the sensitive parameters are shown in Table 4. They are all within ranges found in the BRENDA databank.

## Conclusion

A model simulating *Chlorella protothecoides* cell metabolic behavior under heterotrophic condition and describing metabolic network flux kinetics and energetic states has been developed and calibrated. Simulation results show adequate fit with experimental data. Flux analysis is also in high agreement with literature data. A sustained high lipid synthesis metabolic activity was further confirmed from model simulations with higher lipid flux and lower TCA activity. The model was also used to analyze the dynamic distribution of the carbon source to the main carbon pathways, such as PPP pathway, starch synthesis, lipid synthesis and nucleotides synthesis. In addition, as the model included a high number of parameters, it described not only experimental data, but also most of the metabolic kinetics that showed statistical significance. It can thus be used as an in silico platform for characterizing the cell lines as well as to search for ‘‘optimal’’ culture strategy either by management through rational adjustment of the main nutrient concentrations that affect glucose and/or glycine concentration with time or by genetic manipulation of certain predicted critical enzymes. However, much work remains to be done: it would be of interest to add more metabolic reactions from extracellular multiple nutrients, like ions; and separating the lipids pools to more interest classes; get larger data sets including both extra- and intracellular experimental data, to test and validate the platform as a predictive tool.

## Abbreviations

Abbreviations of metabolites names and parameter symbols were detailed in Tables 1, 2, 3 and 4.

## Additional file

**Additional file 1: Table S1.** Mass balances of state variables in the model.

## Data Availability

All data generated or analyzed during this study are included in this published article.
