# Supplementary material for: A kinetic metabolic study of lipid production in Chlorella protothecoides under heterotrophic condition
Source: Microb Cell Fact. 2019 Jun 28;18:113. doi: 10.1186/s12934-019-1163-4 (PMC6598345; doi:10.1186/s12934-019-1163-4)
Supplement: Supplementary file 1 — Additional file 1: Table S1. Mass balances of state variables in the model. [file 12934_2019_1163_MOESM1_ESM.docx]

**Additional Table S1**

Table S1. Mass balances of state variables in the model

| **No.** | **Mass balances of each metabolite** | **Unit** |
| --- | --- | --- |
| 1 | $\frac{dAA}{dt}=+ V\_GLDH - (V\_growth * AA)$ | *mmol/gDW/day* |
| 2 | $\frac{dADP}{dt} =+ V\_PPRiBP - V\_CK + V\_AK - (V\_growth * ADP)$ | *mmol/gDW/day* |
| 3 | $\frac{dAKG}{dt} =+ V\_ISOD - V\_AKGDH - V\_GLDH - (V\_growth * AKG)$ | *mmol/gDW/day* |
| 4 | $\frac{dATP}{dt} = + V\_CK - V\_AK - (V\_growth * ATP)$ | *mmol/gDW/day* |
| 5 | $\frac{dAcCOA}{dt} = + V\_PDH - V\_CS -(12 * V\_FASN) +(12 * V\_Lipase) -(V\_growth * AcCOA) - (V\_growth\_AcCOA * V\_growth)$ | *mmol/gDW/day* |
| 6 | $\frac{dCIT}{dt} = + V\_CS + V\_MLD - V\_ISOD - (V\_growth * CIT)$ | *mmol/gDW/day* |
| 7 | $\frac{dF6P}{dt} = + V\_GPI - V\_PFK + V\_FBPase +(2 * V\_TK) - (V\_growth * F6P)$ | *mmol/gDW/day* |
| 8 | $\frac{dFUM}{dt} = + V\_SDH - V\_FH - (V\_growth * FUM)$ | *mmol/gDW/day* |
| 9 | $\frac{dG1P}{dt} = + V\_PGM -(25 * V\_ADPG) +(25 * V\_AP) - (V\_growth * G1P)$ | *mmol/gDW/day* |
| 10 | $\frac{dG6P}{dt} = + V\_HK - V\_GPI - V\_G6PDH - V\_PGM - (V\_growth * G6P) - (V\_growth\_G6P * V\_growth)$ | *mmol/gDW/day* |
| 11 | $\frac{dGD}{dt} = +(2 * V\_PFK) -(2 * V\_FBPase) - V\_PGK + V\_TK - V\_TPI - (V\_growth * GD)$ | *mmol/gDW/day* |
| 12 | $\frac{dGlyP}{dt} = + V\_TPI - V\_GPAT - (V\_growth * GD)$ | *mmol/gDW/day* |
| 13 | $\frac{dLipid}{dt} = + V\_GPAT + V\_FASN - V\_Lipase - (V\_growth * Lipid) - (V\_growth\_Lipid * V\_growth)$ | *mmol/gDW/day* |
| 14 | $\frac{dMAL}{dt} = - V\_MLD + V\_FH - V\_ME - (V\_growth * MAL)$ | *mmol/gDW/day* |
| 15 | $\frac{dPEP}{dt} = + V\_PGK - V\_PK + V\_ME - (V\_growth * PEP)$ | *mmol/gDW/day* |
| 16 | $\frac{dPYR}{dt} = + V\_PK - V\_PDH + V\_GHMT - (V\_growth * PYR) - (V\_growth\_PYR * V\_growth)$ | *mmol/gDW/day* |
| 17 | $\frac{dRX}{dt} = + V\_G6PDH -(3 * V\_TK) - V\_PPRiBP - (V\_growth * RX) - (V\_growth\_RX * V\_growth)$ | *mmol/gDW/day* |
| 18 | $\frac{dSCOA}{dt} = + V\_AKGDH - V\_SCOAS - (V\_growth * SCOA)$ | *mmol/gDW/day* |
| 19 | $\frac{dSUCC}{dt} = + V\_SCOAS - V\_SDH - (V\_growth * SUCC)$ | *mmol/gDW/day* |
| 20 | $\frac{dStarch}{dt} = + V\_ADPG - V\_AP - (V\_growth * Starch)$ | *mmol/gDW/day* |
| 21 | $\frac{dEGLC}{dt} = ( - V\_HK ) * X$ | *mmol/L/day* |
| 22 | $\frac{dX}{dt}= V\_growth * X$ | *gDW/L/day* |
| 23 | $\frac{dGLY}{dt} = ( - V\_GHMT ) *X$ | *mmol/L/day* |
| 24 | $\frac{d{CO}_{2}}{dt} = ( + V\_PDH + V\_G6PDH + V\_ISOD + V\_AKGDH + V\_ME ) * X$ | *mmol/L/day* |
